# Exploration of and insights into advanced topical nanocarrier systems for the treatment of psoriasis

**DOI:** 10.3389/fmed.2022.1017126

**Published:** 2022-12-15

**Authors:** Miao Zhang, Seokgyeong Hong, Xiaoying Sun, Yaqiong Zhou, Ying Luo, Liu Liu, Jiao Wang, Chunxiao Wang, Naixuan Lin, Xin Li

**Affiliations:** ^1^Department of Dermatology, Yueyang Hospital of Integrated Traditional Chinese and Western Medicine, Shanghai University of Traditional Chinese Medicine, Shanghai, China; ^2^Institute of Dermatology, Shanghai Academy of Traditional Chinese Medicine, Shanghai, China

**Keywords:** novel drug delivery systems, psoriasis, topical therapy, transdermal, nanocarriers

## Abstract

Psoriasis is a chronic inflammatory skin disease with an underlying autoimmune pathogenesis that has brought great distress to patients. Current treatment options include topical therapy, systemic therapy, and phototherapy. By disrupting the stratum corneum, nanocarriers have unique advantages in allowing drug carriers to be tailored to achieve targeted drug delivery, improve efficacy, and minimize adverse effects. Furthermore, despite their limited success in market translatability, nanocarriers have been extensively studied for psoriasis, owing to their excellent preclinical results. As topical formulations are the first line of treatment, utilize the safest route, and facilitate a targeted approach, this study, we specifically describes the management of psoriasis using topical agents in conjunction with novel drug delivery systems. The characteristics, advantages, weaknesses, and mechanisms of individual nanocarriers, when applied as topical anti-psoriatic agents, were reviewed to distinguish each nanocarrier.

## 1 Introduction

Psoriasis is a recurrent inflammatory autoimmune skin disorder that ranges in severity from highly inflammatory red erythema patches supported by silvery scales to the entire skin surface, exacerbating itching, irritation, and body pain (1). The etiology of psoriasis involves a combination of hereditary and environmental factors that lead to histological changes in the skin (2, 3). At present, the treatment and management of psoriasis mainly involve topical agents, phototherapy, and systemic therapy. Topical agents are generally recommended as first-line treatment for mild psoriasis. However, for more severe conditions, phototherapy or systemic therapy is more suitable than topical agents (4). Although these methods can lessen the symptoms and signs of psoriasis, they all have limitations and are unable to completely cure the disease (5–7).

Therefore, recent research has attempted to design and develop a novel drug delivery system to compensate for the deficiencies of traditional drug delivery systems. The nanotechnology-based delivery systems have shown great advantages in increasing drug concentration at the targeted site, drug encapsulation efficiency, and skin penetration, thereby providing better efficiency, reduced side effects, and improved patient compliance. This review examines the various challenges in treating psoriasis, contemporary research into new drug delivery systems, and future potential for therapy.

## 2 Core pathogenesis: Massive proliferation of keratinocytes

The main clinical manifestation of psoriasis is evident in the outermost layer of the skin, which comprises keratinocytes. Keratinocytes were initially thought to be the primary cause of infection, with the immune system resulting in an excessive proliferation of skin cells, leading to immune-mediated disorders (8). Psoriasis occurs when skin cells accumulate in the epidermis instead of shedding, causing visible lesions (9). Additionally, the recognition of antimicrobial peptides is closely related to dendritic cell (DC) activation. LL-37, the most studied psoriasis-associated antimicrobial peptide, binds to DNA and stimulates toll-like receptors in plasmacytoid DC (10). Subsequently, plasmacytoid DC activation produces type I IFN (IFN-α and IFN-β), which further promotes the phenotypic maturation of myeloid DC. Th1 cells then differentiate and together with Th1 cytokines secrete a mass of inflammatory cytokines, of which the most pivotal are IL-12 and IL-23 in psoriasis. Subsequently, IL12 stimulates Th1 to produce IFN and TNF-α and IL23 stimulates Th17 to produce IL17 and IL22 (11–13).

The maintenance stage of psoriatic inflammation is the proliferation of keratinocytes, mainly involved in four aspects. The most important is the activation of adaptive immune responses by T-cell subsets, represented here by the involvement of Th17 cytokines, such as IL-23, IL-22, and IL-17 (14), followed by the inflammatory milieu activating keratinocyte proliferation through IL-17, TNF-α, and IFN-γ (10). However, LL37 and DNA greatly increase the type I IFN production. Moreover, with the help of cytokine (IL-1, IL-6, and TNF-α), chemokine, and AMP secretion, keratinocytes can also participate actively in the inflammatory cascade.

**FIGURE 1:**
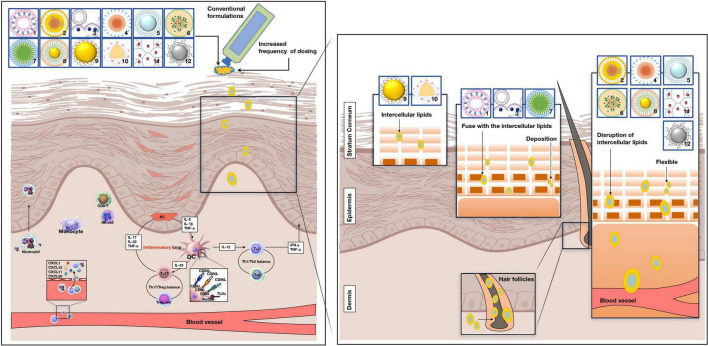
Potential penetration routes of novel drug systems and conventional formulations. Pathogenesis of psoriasis is related to the KC/DC/T loop. 1: liposomes, 2: nanostructured lipid carriers, 3: niosomes, 4: nanoemulsions, 5: ethosomes, 6: solid lipid nanoparticles, 7: micelles, 8: gold particle, 9: nanocapsules, 10: nanosphere, 11: dendrimers, 12: sliver particle. pDC, plasmacytoid dendritic cells; DC, dendritic cells; KC, keratinocyte. NK -cell, natural killer cell; IL-17A, interleukin-17A; IL-17 F, interleukin-17 F; IL-17, interleukin-17; IL-12, interleukin-12. TNF-α, tumor necrosis factor-α; IL-6, interleukin-6; IL-1β, interleukin-1β; IFN-γ, interferon-γ; Th1 cell, T helper 1 cells; Th2 cell, T helper 2 cell; Treg cell, T regulatory cells.

Previous studies have shown that the TNF-α and IL-23/IL-17A axis is the main driver of psoriasis, and the interaction between IL-17A and keratinocytes is a key issue in developing psoriasis. This review aimed to highlight the important role of keratinocytes in the pathogenesis of psoriasis because patients with psoriasis are typically characterized by epidermal hyperproliferation, which aggravates the burden of traditional drug administration. Furthermore, we will present the unique advantages of the novel drug delivery system by breaking through the thick stratum corneum to explore the treatment of psoriasis patients (Figure 1).

## 3 Treatment challenges: Difficulties in penetrating the horny psoriatic layer

The function of the skin in psoriasis and how the pathological condition of the skin minimizes or exceeds the barrier function of the cuticle and epidermis have been a concern for researchers for many years. An experimental study in Japan reported that psoriatic skin has lower water content, free fatty acids, and natural moisturizing factors than normal skin, while the sebum content remains unaffected (15). The main concern regarding an effective response to dermatological agents is their need to reach the targeted site and remain at effective concentrations for a period. Abnormal thickening of the stratum corneum (SC) results from the hyperproliferation of keratinocytes and as a direct result of hindered penetration of the skin; topical drug delivery to psoriatic skin presents a difficult challenge for conventional formulations.

## 4 Conventional management for psoriasis: The multi-organ pitfalls remains

**FIGURE 2:**
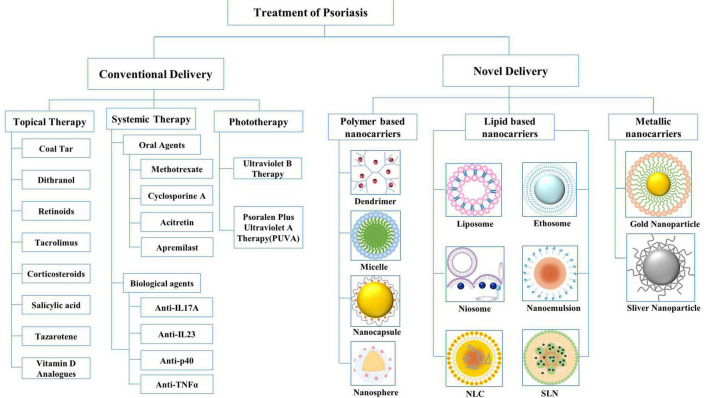
Conventional drug delivery systems and novel drug delivery systems for the treatment of psoriasis. NLC, nanostructured lipid carriers; SLN, solid lipid nanoparticles.

Treatment choices for psoriasis management comprise three main approaches: topical treatment, systemic treatment, and phototherapy (Figure 2A). Topical therapy is generally considered the first-line treatment for mild-to-moderate localized psoriasis and can be used with phototherapy or systemic therapy for moderate-to-severe psoriasis. Topical agents used include coal tar, dithranol (DIT), and retinoids, as well as tacrolimus (TAC), corticosteroids, salicylic acid (SA), tazarotene, and vitamin D analogs. However, owing to the lack of continuous elimination of the lesion, this may relapse and increase the psychological burden of the patient (16, 17).

Oral retinoids, methotrexate (MTX), cyclosporine (CYC), and fumaric acid esters are utilized in the treatment of psoriasis. Organ toxicity may occur after systematic treatment with these drugs, such as the fact that MTX is toxic to the liver and cyclosporine is nephrotoxic (18, 19). Biological agents, specifically anti IL-17A, anti-IL-23, and anti p-40 agents, have been shown to be effective against psoriasis in recent years. However, as anti-IL17A agents are associated with mycosis and neutropenia, anti-TNF-α treatments are not recommended for patients with heart problems (20, 21).

Compared with biological agents, apremilast (a small-molecule inhibitor of phosphodiesterase 4) acts at an earlier point in the inflammatory cascade, resulting in broad regulation of multiple inflammatory mediators (22). As for the conventional phototherapies, narrow-band UVB and photochemotherapy, such as PUVA (psoralen plus UVA), are used to a lower extent. Therefore, current research aims to apply novel nanocarriers to safely and effectively delivery anti-psoriatic drugs. Thus, we decided to restrict this study to topical approaches.

## 5 Promising strategies: Advanced topical nanocarrier systems

Nanoparticles offer advantageous properties as alternatives to conventional formulations. Therefore, by adding a targeting moiety, controlling the size of the vector, and combining hydrophobic drugs in hydrophilic carriers, the carrier can be customized. An ideal drug carrier should cross the thick stratum corneum and selectively recognize target cells by surface ligands, and most importantly, the drug-ligand complex must be stable in the biological environment and the drug carrier must be biodegradable and non-toxic (23). Novel drug delivery systems can be classified into three broad categories based on their main components: polymer-based, lipid-based, and metallic nanocarriers (Figure 2B). In this review, we discuss the above-mentioned nanocarriers, which have been proven effective in clinical and experimental studies in the past few years. The advantages and disadvantages of nanocarriers for psoriasis treatment are summarized in Table 1.

**TABLE 1:** The advantages/disadvantages of nanocarriers for the treatment of psoriasis.

Nanoformulation	Advantages	Disadvantages
Liposomes	Biocompatible, ease of surface alteration and amphiphilic nature	Weak loading capacity, rapid drug leakage, limited physical and chemical stability during storage

Ethosomes	The ability of targeting deep skin layers, showing excellent flexibility, and deformability	Risk of organic solvent residue, poor yield

Niosomes	Improving the bioavailability of insufficiently absorbed drugs and possessing more penetration power of drugs through skin	Less skin penetration, do not reach up to deeper skin layer

Nanoemulsions	Elastic properties and fluid performance	Usage of expensive instruments

Solid lipid nanoparticles	Higher efficacy, biocompatible, biodegradable, flexibility of size, and surface manipulation	Poor stability, poor batch to batch reproducibility, sterilization difficulties, low drug loading

Nanostructured lipidic carriers	Biodegradable, increasing the drug payload and reducing drug leakage during storage	Sterilization difficulties

Micelles	Thermodynamic stability, self-assembling, and targeting potential	Not good for hydrophilic drugs

Dendrimers	Ease of preparation and modification; better skin penetration	Polymer dependent biocompatibility

Nanocapsule	Sustained release, incremental drug selectivity and effectiveness, improvement of drug bioavailability, and alleviation of drug toxicity	Physical and chemical instability

Nanosphere	Lower polymer content and a high loading capacity, significant drug accumulation	Low stability

Gold nanoparticle	Ease of functionalization, large surface area compared to volume and small particle size	Cytotoxicity to epidermal keratinocytes and hair follicles’ stem cells, causing skin irritation

Sliver nanoparticle	Targeting, improving solubility and stability, hindering side effects	Whether it passes the skin barrier or remains retained in the skin layers was controversial

### 5.1 Mechanism of action of nanostructures

Lipid-based nanocarriers improve skin surface adhesion, contact with SC, and boost the penetration efficiency of biologically active ingredients into the skin (24). Polymer-based nanocarriers provide the facility of enhanced concentration gradient at the skin surface and offer tunable physical or chemical properties that can achieve sustained or controlled release of the incorporated drug at the delivery site (25). Metallic nanocarriers are conjugated to diverse targeted peptides for functional nanoparticles to penetrate biological tissues successfully (26). The potential penetration routes of the novel drug delivery system are shown in Figure 1.

### 5.2 Lipid based nanocarriers

Lipid-based nanocarriers comprise physiological lipids whose structures are safe and non-toxic. These mainly include the following six types: liposomes, ethosomes, niosomes, nanoemulsions (NEs), solid lipid nanoparticles (SLNs), and nanostructured lipidic carriers (NLCs).

#### 5.2.1 Liposomes

Liposomes are simple, self-assembling spherical vesicles consisting of one or more lipid bilayers arranged in concentric circles and enclosing equal volumes of aqueous compartments, similar to bilayer membranes of living cells (27). Liposomes are widely used as drug transporters for lipophilic and hydrophilic molecules because of their amphiphilic nature and bilayer structure (28). To evaluate the anti-psoriasis effects of cationic liposomes, dioleoyl-3-trimethylammonium propane (DOTAP) was used to prepare the CYC-loaded liposomes. The results demonstrated a strong affinity for anionic skin membranes, resulting in improved efficacy in an imiquimod-induced psoriatic plaque model (29). Another study showed that peptide-modified curcumin-loaded liposomes assisted in skin permeation and drug retention of liposomes. Consequently, peptide-modified curcumin-loaded liposomes significantly improved psoriatic lesions and decreased epidermal thickness (30). These findings proved that various modifications of liposomes could improve skin permeability. However, there is no definitive evidence of its effectiveness in patients with psoriasis, as it seems to accumulate a majority of drugs in the upper layers of the SC. One study addressed this deficiency by designing capsaicin and anti-TNF-α small interfering RNA-encapsulated cyclic cationic head lipid-polymer hybrid nanocarriers. Based on *in vitro* skin distribution studies, this complex effectively delivered FITC-siRNA up to 360 μm skin depth, suggesting that anti-TNF-α small interfering RNA and capsaicin can be delivered into the deeper dermal milieu with the cooperation of novel cationic lipid-polymer hybrid nanoparticles (31).

#### 5.2.2 Ethosomes

Ethosomes are novel ultra-deformable nanovesicles with high-quality alcohol in water, comprising phosphides that are generally used at a concentration of 0.5–10% (32). Interactions with the polar head group of lipids enable fluidity of the skin tissue structure lipid bilayer, which triggers drug release into deeper layers of the epidermis. One study compared the safety and clinical efficacy of liposomes and ethosomes in psoriasis patients. After treatment, the mean psoriasis area and severity index change for liposomes and ethosomes were −68.66% and −81.84%, respectively, indicating that ethosomal preparations were more effective than liposomes (33). In another study, ethosomal gel loaded with MTX-incorporated SA was assessed for its anti-psoriatic potential in imiquimod-induced rice. Based on the results obtained from continuous monitoring for 24 h, MTX-SA-ethosome gel releases MTX slowly over an extended period, with >30% drug retention detected in the skin (34). These studies have confirmed the synergistic effect of incorporating alcohol into liposomes. However, the risk of allergies may concomitantly increase with an increase in the amount of ethanol. Along with safety concerns, the ethanolic core of ethosomes may evaporate at relatively high temperatures in the local inflammatory area of psoriatic skin. Both issues require further pharmacodynamic investigation and clarification of their clinical efficacy.

#### 5.2.3 Niosomes

Niosomes self-assemble from amphiphilic non-ionic surfactants with cholesterol and are oriented into a bilayer structure with a neutral total charge (35). The presence of non-ionic surfactants modifies the horny layer of psoriasis into looser, more permeable tissue; therefore, the residence time and local concentration of the drug in the SC and epidermis can be improved (36). Niosomes containing MTX in chitosan gels had maximum penetrability, with a significantly reduced total score, from 6.2378 ± 1.4857 to 2.0023 ± 0.1371, compared with commercially available gels (37). This study was combined with other experimental results, which demonstrated that MTX-encapsulated noisomes exhibited noticeable changes in ameliorating skin lesions, decreasing spleen index and epidermal thickness, and downregulating the mRNA expression of proinflammatory cytokines (38). In addition, celastrol niosomes offer an enhanced *ex vivo* osmotic profile of drug deposition in viable skin layers, and a significantly higher degree of keratosis, reduction in drug activity, and epidermal thickness were achieved (39). In the literature review, we noted that the composition, size, and surface charge of niosomes play important roles in determining the permeability, stability, and retention of drugs in the skin layer.

#### 5.2.4 Nanoemulsions

Nanoemulsions are formed by dispersing two immiscible liquids (frequently water and oil) with droplet sizes ranging from 20 to 500 nm (40). Three types of NEs have been well-studied for particular applications: oil-in-water (o/w) emulsions, water-in-oil (w/o) emulsions, and bicontinuous NEs. The effectiveness of transdermal drug absorption of w/o NEs is promoted *via* skin appendages by increasing the solubility of drugs and producing a larger concentration gradient between NEs and the skin (41). Similarly, to promote the permeation of CYC into the SC, one study introduced a nanoemulsion for transporting cyclosporine as an anti-inflammatory drug with the addition of nutmeg and virgin coconut oil mixture. The emulsified CYC confirmed not only an increase in drug loading but also increased skin hydration in wholesome volunteers due to the greater content of fatty acids in the combined oil solution (42). Moreover, some specifically designed NE systems have been recommended as moisturizers for daily skin care of patients with psoriasis. Therefore, scholars developed o/w NEs using rice bran oil, and the results confirmed that elevated NEs maintained normal skin pH and hydration (43). However, the low viscosity of NEs may also be overcome through a hydrogel thickening process, limiting its topical application.

#### 5.2.5 Solid lipid nanoparticles

Unlike conventional liposomes, SLNs are solid lipids with an average diameter of 10–1000 nm that remain solid at room and body temperatures (44). There are two main factors that affect drug release characteristics: drug deposition patterns and the melting points of lipids. The beneficial effects of SLNs can be summarized as the “occlusion effect,” which occurs because of the formation of an occlusive hydrophobic film on top of the SC, resulting in better hydration and transcutaneous penetration (35). One study verified that betamethasone dipropionate (BD)- calcipotriol (CT)-loaded SLNs extensively increased the dermal absorption of BD/CT and delayed the abrupt growth of keratinocytes. Consistently, an *in vivo* mouse tail model demonstrated that the administration of BD-CT-SLNs reduced epidermal thickness and increased melanocyte count without side effects compared to Daivobet™, which can verify the anti-psoriatic activity of BD-CT-loaded SLNs (45). Similar observations were reported when SLNs were applied as a topical solution containing a combination of MTX and etanercept (46). These results show that SLNs have great potential to limit dose-dependent toxicity by reducing the systemic exposure of the encapsulated drug.

#### 5.2.6 Nanostructured lipidic carriers

Nanostructured lipidic carriers were designed to fill in the gap between SLNs regarding drug solubility and leakage by replacing up to 30% of the solid lipid mass of SLNs with liquid lipids. Although a certain amount of liquid lipids is replenished, the output of NLCs by mixing remains in a solid form at room or body temperature (47). When incorporating liquid lipids (an imperfect and less-ordered crystalline structure of NLCs) was created, displaying a considerable “occlusion effect,” which is appropriate for topical administration. A comparative study evaluated the potential use of carbomer gel-bearing methotrexate-loaded NLC carriers for topical application of methotrexate in contrast to SLNs. The skin drug deposition study indicated the greatest deposition of drug-enriched NLC5 hydrogel (28.8%), in contrast to plain drug-enriched hydrogel (11.4%) and drug-enriched SLN hydrogel (18.6%) (48). Fluorescence microscopy showed that these lipid-based systems have a localization effect in deeper skin regions. Another randomized controlled trial further confirmed the anti-psoriasis effects of NLCs by formulating and characterizing acitretin (ACT) NLCs. A notably higher deposition of acitretin was discovered in human cadaver skin from ACTNLC gel (81.38 ± 1.23%) than from ACT plain gel (47.28 ± 1.02%) (49).

### 5.3 Polymer-based nanocarriers

Polymer-based nanocarriers are colloidal structures composed of macromolecules that contain natural or synthetic polymers as the chief excipients. Compared with lipid-based nanocarriers, polymers tend to accumulate in the dermis layer, suggesting that the former is suitable for topical treatment, whereas the latter is more appropriate for transdermal treatment (50). Four types of polymer-based nanocarriers, namely, micelles, dendrimers, nanospheres, and nanocapsules, are discussed in the next section.

#### 5.3.1 Micelles

Micelles are spherical structures with diameters less than 100 nm, formed by the self-assembly of amphiphilic molecules in an aqueous system through a hydrophobic effect (51). In the micelle structure, the hydrophobic core carries lipophilic drugs and the hydrophilic shell carries hydrophilic drugs, which are suitable for drug administration. To enhance the cutaneous bioavailability of TAC, TAC-loaded polymeric micelles were formulated using biodegradable and biocompatible methoxy-poly (ethylene glycol)-dihexyl-substituted polylactide diblock copolymer. The results showed that compared with Protopic, TAC deposition in skin was significantly increased by this formula (0.1% W/W; TAC ointment, 1.50 ± 0.59 and 0.47 ± 0.20 μg/cm, respectively) (52). In a similar way, silibinin-loaded polymeric micelles were prepared in another study. The average particle size of the optimized samples was 18.3 ± 2.1 nm, the encapsulation efficiency was 75.8 ± 5.8%, and the release time of silybin was prolonged. Moreover, silibinin permeation through psoriatic skin after 48 h of treatment with polymeric micelles and the aqueous control was 80.35 and 92.6, respectively (53). These micelles have been designed for targeted drug delivery because of their easy structural deformation under the influence of pH, temperature, or reduction-oxidation reactions *in vivo*. Therefore, micelles are more suitable for local administration or in combination with other polymer materials when controlled drug release is required (54).

#### 5.3.2 Dendrimers

Dendrimers are highly-branched and nano-sized micromolecules with a controlled, globular, reactive three-dimensional structure. Drug molecules form drug-dendritic molecular conjugates through non-covalent interactions or covalently linked functional groups (55). The three dendrimer types were peptide dendrimers, glycodendrimers, and lysine-core dendrimers. One study explored the potential of DIT-loaded polypropylene imine (PPI) dendrimers and their characterization was performed using spectroscopy and transmission electron microscopy. Confocal laser scanning-microscopy images and skin penetration studies demonstrated that PPI can be utilized to enhance the local bioavailability of molecules in a controlled manner. PPI enhances transdermal absorption in a controlled manner, while DIT accumulation in the skin *via* dendritic molecular vectors may help optimize drug targeting to the epidermis and dermis (56). Another study focused on the enhancement of transdermal delivery of 8-methoxyp-soralen (8-MOP) DOTAP by the dendritic molecules G3 and G4 of polyamidoamine (PAMAM). Another study focused on the enhancement effect of PAMAM dendrimers G3 and G4 on transdermal delivery of 8-MOP. Enhanced *in vivo* 8-MOP skin penetration into the deeper layers of the skin was obtained, and G4 PAMAM dendrimers provided a better penetration enhancement effect for 8-MOP compared to G3 PAMAM (57). Moreover, one study compared the PAMAM dendrimers’ suitability with DOTAP liposomes for topical delivery of siRNA against TNF-α. The results showed that phenotypic and histopathological features were improved, and IL-6, TNF-α, IL-17, and IL-22 levels decreased in the dendritic plexus and liposome treatment groups compared to the imiquimod group (58).

#### 5.3.3 Nanocapsule

Nanocapsule is a type of nanoparticle that consists of one or more active components (core) and a protective matrix (shell), with the core consisting of a liquid suspension containing the medicine and the polymer constituting the shell (59). One study created tretinoin-loaded nanocapsules and assessed the influence of nanoencapsulation on the photostability of tretinoin from different perspectives. Inferring that nanocapsules are a suitable carrier for tretinoin to treat psoriasis, photodegradation experiments showed a 2-fold increase in drug stability in a methanolic solution, with an increased half-life between 85 and 100 min (60). In another experimental investigation, the stability of DIT-loaded lipid-core nanocapsules was compared to that of medication-free solutions by photodegradation against UVA light. It showed greater stability (half-life times of approximately 4 and 1 h for the DIT-loaded lipid-core nanocapsules), and an irritation test was conducted to evaluate the safety of the formulations; it was proved that the drug’s toxicity was reduced due to nanoencapsulation (IS = 0) compared to the effects observed with DIT dispersion (10.43 ± 0.67) (61). Vertical Franz diffusion cells were used to study and test the dexamethasone-loaded polymeric nanocapsules. The results of the *in vitro* release showed that there was minor drug release per cm2 after 2 and 24 h (62).

#### 5.3.4 Nanosphere

Nanospheres are matrix systems in which medicine is either disseminated inside the polymer matrix or adsorbed on the surface of the sphere. The polymer matrix constituents and capacity to absorb fluid affect the speed of drug release (63). Nanospheres have received considerable attention recently, owing to their protective shell and ability to oxidize easily. Previous study created polymeric betamethasone disodium 21-phosphate (BP) nanoparticles and PEG-block-PLA/PLGA copolymers and homopolymers from poly (D, L-lactic acid) and poly (D, L-lactic/glycolic acid). Small nanoparticles released more BP than larger ones, whereas PLGA homopolymers released BP more quickly than PLA homopolymers (64). Tyrosine-derived nanospheres (TyroSpheres) encapsulated in anti-proliferative paclitaxel were further developed. The findings demonstrated that TyroSpheres increased [approximately 4,000 times better than that of phosphate-buffered saline (PBS)] and permitted sustained, dose-controlled release over a 72 h period under conditions simulating skin permeation. By enabling paclitaxel to be delivered into the epidermis at concentrations of >100 ng/cm^2^ of skin surface area and by increasing the cytotoxicity of loaded paclitaxel to human keratinocytes, tyrospheroids may effectively treat psoriasis (65).

### 5.4 Metallic nanocarriers

Metallic nanomaterials have been of significant interest to the scientific community for many decades. These particles have antibacterial, antifungal, and anti-skin cancer effects, and therefore, are promising for the management of dermatological diseases. Types of metallic nanoparticles have been synthesized using different elements, such as gold nanoparticles (AuNPs) and silver nanoparticles (AgNPs).

#### 5.4.1 Gold nanoparticles

Solid colloidal particles, called AuNPs, with sizes ranging from 1 to 100 nm, are created from metal precursors. Nanoparticles and their contents can also pass through biological barriers that are difficult to access and penetrate, owing to their small size. Using different chemistries or because of their high affinity for thiolated molecules, they can function easily with all types of electron-donating compounds (26, 66). The ease of functionalization, increased surface area to volume ratio, small particle size, and the anti-inflammatory action they perform provide a synergistic effect when loaded with anti-inflammatory drugs (67). Combining topical AuNPs and AgNPs with cornus mas inhibits proliferation in human plaque psoriasis by downregulating the activity of nuclear factor-3B, as well as decreasing cluster of differentiation 68-positive macrophages and IL-12 and TNF-α production (68). In another study, woodfordia fruticosa (flower extract)-enriched AuNPs were used to reduce hyperplasia, parakeratosis, and serum concentrations of TNF-α, IL-22, and IL-23 and showed that 1% AuNPs (ointment) mixed with Swiss albino mice showed the lowest energy level and had a significant therapeutic value (69).

#### 5.4.2 Silver nanoparticles

Silver nanoparticles are one of the most promising metal nanoparticles and have been widely used in nanomedicine, especially for the diagnosis and treatment of cancer. In addition, the potential of AgNPs for the delivery of antimicrobial, antibacterial, antifungal, and anti-material agents has been explored. Moreover, AgNPs have been effectively used as delivery carriers for anti-psoriasis drugs (70). A team prepared biocompatible AgNPs containing fruit extracts of European blackberry and examined their anti-inflammatory effects. The synthesized nanoparticles have good anti-inflammatory effects and have been studied both *in vivo* and *in vitro*. *In vitro*, anti-inflammatory effects were shown by a reduction in cytokine production and maintenance of low levels after UVB irradiation. *In vivo*, AgNPs pre-administration reduced cytokine levels in the foot tissue and had a long-term protective effect. AuNPs are considered promising anti-psoriasis treatments (71). Another study evaluated the anti-inflammatory capability of nanoparticles created in rats using carrageenan-induced hind foot edema models and albumin denaturation and suggested that silver nanoparticles may reduce or prevent the release of acute inflammatory mediators. This study unequivocally demonstrated that Selaginella myosurus mediated by AgNPs might be a source of anti-inflammatory medications (72).

In addition to the above-mentioned nanocarriers, diverse novel carriers have been developed for the effective delivery of various anti-psoriatic drugs. Table 2 lists the topical applications of nanocarriers in drug delivery for psoriasis therapy, which collectively constitutes a large category of human conditions. Novel delivery systems have gained a unique position for safe and effective drug delivery to other dermatological diseases, including atopic dermatitis, melanoma, and acne. Considering the unique features of each nanocarriers, we suggest that a proper combination should be considered based on the physicochemical properties of the loaded drugs and the clinical characteristics of patients with psoriasis.

**TABLE 2:** Topical applications of nanocarriers in drug delivery for psoriasis treatment.

Nanocarriers	Typical components	Carried drug	Preparation technique	Excipients	Size (nm)	Encapsulation efficiency (%)	Drug release (%)	Skin permeability	Stability	Experin- mental studies	Transdermal delivery mechanism	References
												
					Zeta potential (mV)							
		Cyclosporine	Ethanol injection, thin film hydration, reverse phase evaporation	N-(1-(2,3-dioleoyloxy) propyl)- cholesterol, chloroform, ethanol	111 ± 1.62 41.12 ± 3.56	93 ± 2.12	120 h:43.86 ± 4.85	NN/A	4 (4^°^)	IMQ rats	(1) Improve the hydration degree of the SC. (2) Change the structure of the epidermis by fusion with the SC and disrupt its lipid arrangement. (3) Permeate into the intercellular spaces *via* diffusion and capillary action	(19)
**Liposomes**	phospholipids											
		Curcumin	Thin film hydration	Peptide	94–100 −22.0	97	96 h:>80	Permeate into the dermis	1 (4^°^)	IMQ rats		(20)
		
		Capsaicin SiRNA	Double emulsion solvent evapo-ration	Ethanol	163 ± 9 35.14 ± 8.23	92	N/A	Permeate into deep dermis	N/A	IMQ rats		(21)

		Anthralin	Thin film hydration	Cholesterol/PL-90G, chloroform and methanol	146–381 N/A	85.0 ± 0.6	27.1 ± 0.4	Permeate through the upper layers of the SC	N/A	Psoriasis patients	The flexibility and deformability of ethosomes facilitate drugs passing through SC and target deep skin layers	(23)
**Ethosomes**	Phospholipids (0.5–10%), ethanol (20–45%)											
		Methotrexate Salicylic Acid	Cold method	Soya phosphatidyl-choline, chloroform, methanol, hydro-ethanolic solution	376.04 ± 3.47 ∼20	91.77 ± 0.02	26.13 ± 1.61	8 h:5.87 ± 0.01% Permeate through the SC	N/A	IMQ rice		(24)

		Methotrexate	Lipid layer hydration	Glycerin, propylene glycol	N/A	N/A	N/A	N/A	N/A	Psoriasis patients	(1) The bilayer membrane made of non-ionic surfactant containing cholesterol has strong permeability. (2) High chemical stability	(27)
**Niosomes**	Non-ionic surfactants cholesterol											
		Methotrexate Nicotinamide	Ethanol injection	Absolute ethanol	181.27 ± 1.44 −24.53 ± 1.37	71.05 ± 0.8	N/A	Permeate into the dermis, mainly in the SC	N/A	BALB/c mice		(28)
			
		Celastrol	Thin film hydration	Cholesterol, carbopol 934, span 20, span 60	147.4 ± 5.6 textbf −48.9 ± 1.1	N/A	N/A	N/A	N/A	IMQ rice		(28)

		Cyclosporine	Emulsification	Tween 80	159.9 –	N/A	3 h:81.49	N/A	>3 months (4^°^/RT)	Healthy volunteers	(1) Increase the solubility and diffusivity of SC. (2) Extract and swell skin lipids to enhance penetration through the pores. (3) Permeate the scaly keratinized psoriatic skin through the hydrophilic pathways and pores	(32)
**Nano-emulsions**	Oil phase, surfactant and cosurfactant											
		Rice bran oil	Emulsion phase inversion	N/A	69 ± 17 –	N/A	N/A	N/A	>90 days (4°/RT)	Healthy volunteers, psoriasis patients		(33)

		Psoralen	Solvent injection	Precirol ATO 5, oleic acid, tween 80, soybean phospholipids	296.6 ± 49.5 −40.0 ± 5.9	N/A	∼70	Low permeation in hyperproli- ferative skin	N/A	Nude mice	(1) Fusion with membrane. (2) Lipid-fluidizing property. (3) Occlusive effect. (4) Utilizing the skin transport pathways, including transcellular route, intercellular route and trans-appendageal route	(35)
			
**SLNs**	Solid lipids	Betametha- sone Calcipotriol	Hot melt high shear homogeni- zation	Compritol 888 ATO, glyceryl monostearate and precirol ATO 5	188 ± 16 N/A	85.10 ± 2.02 (BD) 97.87 ± 0.08 (CT)	48 h:45–56 (BD), 25–31 (CT)	4 h: Permeate into the dermis through appendageal pathway and intercellular route	12 (RT)	HacaT, mouse tail model		(36)
			
		Methotrexate Etanercept	Hot ultrasonication	Cetyl palmitate, Polysorbate	356 ± 2 -27 ± 4	88 ± 2	52 ± 4	8 h:75–80%, Permeate into the dermis	8 (RT)	Psoriatic skin		(37)

		Methotrexate	Solvent diffusion	Glyceryl monostearate, ethanol, acetone, PBS	221 ± 14 −33.6 ± 1.2	62.72 ± 0.94	N/A	24 h: 24.7% ± 2.3%, Permeate through the dermis	16 (RT)	Albino rats	(1) Occlusive effect of solid matrix. (2) Liquid lipids increase skin hydration	(39)
**NLCs**	Solid and liquid Lipids											
		Acitretin	Solvent diffusion	Oleic acid, precirol ATO 5, tween 80, acetone	223 ± 8.92 −26.4 ± 0.86	63.0 ± 1.54	30 h:80.22 ± 3.40	N/A	N/A	Psoriatic patients		(40)

		Tacrolimus	Solvent evaporation	Acetone, phosphate, acetic acid	52.9	88.14 ± 0.20	1.5	Permeate into upper dermis	7 months (4^°^)	Human skin	Preferentially deposited in skin wrinkles, between the corneocyte clusters, where there is a more permeable zone, which could increase drug delivery	(45)
**Micelles**	Surfactant, macromolecule polymer											
		Silibinin	N/A	Cholesterol, lecithin, oleic acid, polox-amer	18.3 ± 2.1	75.8 ± 5.8	4 h:21.8	48 h: 80.35 ± 3.37%, Permeate through the full-thickness psoriatic skin	>3 months (RT)	IMQmice		(46)

		Dithranol	Divergent method	Ethylenediamine	8 ± 0.04 12.0 ± 0.42	57.1 ± 1.32	N/A	24 h: 95.33%, Permeate through skin	N/A	Rats	(1) *In vivo* degradation of covalent bond between drugs dendrimer by suitable enzymes. (2) Releasing of the drug from dendrimer due to physical changes or stimulus like pH, temperature, etc	(48)
			
		8-methoxy-psoralen	Convergent method	Polyamidoamine (PAMAM) dendrimers G3 and G4	N/A	N/A	N/A	29 h: 0.6 mol ^h–1^ cm^–2^	N/A	Wistar rats		(49)
**Dendrimer**	Core, repeating units, terminal surface groups											
		TNF-α siRNA	N/A	PAMAM dendrimer (P-G3), triton X-100, diethyl pyro carbonate water	99.80 ± 1.80 13.40 ± 4.84	98.72 ± 2.02	N/A	N/A	N/A	Wistar rats		(50)

		Tretinoin	Interfacial deposition of the preformed polymer method	Sorbitan monooleate, polymer, acetone	228 ± 08 −7.27 ± 0.66	>99.9%	N/A	N/A	1 h:56–57%	Photo- degradation studies	(1) The initial burst effect: either to surface adsorbed drug either to surface adsorbed drug. (2) The second slow phase: the diffusion of the drug molecules and the reservoir core. (3) Polymer erosion.	(52)
**Nanocapsule**	Active materials (core) and a pro- 679 tective matrix (shell)											
		Dithranol	Interfacial deposition	Ethylene- diaminetetraacetic acid, sorbitan monostearate, Tween 80	241 ± 4, −7.6 ± 0.6	N/A	24 h:96	N/A	15 days: 52%	Photo- degradation studies		(53)
			
		Dexametha- sone	Interfacial deposition	Sorbitan mono-oleate, polysorbate 80	201 ± 06 −5.73 ± 0.42	N/A	N/A	N/A	N/A	Allium cepa root meristem model		(54)

		Paclitaxel	Ultra- centrifugation and resuspension	Suberic acid, poly (ethylene glycol) monomethyl ether, Tween-80	N/A	68 ± 90 (BE) 65 ± 78 (LE)	72 h:8.4–58%	Significant amounts of paclitaxel into the epidermal layer of skin	8 days (4^°^C)	Human cadaver skin	(1) The erosion of polymer matrix. (2) enzymatic degradation of polymeric bonds. (3) Diffusion of the drug entrapped physically	(56)
**Nanosphere**	Spherical shape polymeric matrix											
		Betametha- sone disodium 21-phosphate	Oil-in-water solvent diffusion	Poly (d, l-lactic acid), poly (d, l-lactic/glycolic acid)	178 ± 18	11.7 ± 0.8	1 h:63	N/A	24 h:45%	N/A		(57)

		Methotrexate	Chemical synthesis	Deionized water	4 ± 1 −32 ± 1	N/A	1 h: 80% 24 h: 95%	Observed both in the epidermis and, at less intensity, also in the dermis	6 months (4^°^C)	Wild-type mice Healthy human	Conjugated to various cellular targeting peptides to provide functional nanoparticles	(60)
**Gold nanoparticles** **(AuNPs)**	Gold precusor											
		Cornusmas	Green synthesis	N/A	N/A	N/A	N/A	N/A	N/A	Psoriatic patients		(61)
			
		Woodfordia fruticosa	Biogenic synthesis	Milli-Q water	10∼20 −26.2	N/A	N/A	N/A	N/A	Healthy albino mice		(62)

		Black elderberry fruits	Green synthesis	Resin, absolute ethanol	20∼80 −20.9	N/A	N/A	N/A	1 month	HaCaT cells Wistar rats	AgNPs are up taken by the cells by active mechanisms e.g., endocytosis or by passive mechanisms e.g., by diffusion	(64)
**Silver particles** **(AgNPs)**	Silver precusor											
		Selaginella myosurus plant	Biological synthesis (centrifugation)	N/A	N/A	N/A	N/A	N/A	N/A	Wistar albinos rats		(65)

N/A, not available; IMQ, imiquimod; siRNA, small interfering RNA; SC, stratum corneum; RT, refrigeration ton; BD, betamethasone; CT, calcipotriol; TNF-α, tumor necrosis factor-α; PAMAM, polyamidoamine; PBS, phosphate buffered saline.

## 6 Conclusion and future perspective

Although psoriasis cannot be permanently treated, controlling its clinical manifestations can significantly improve the quality of life of patients and eliminate their psychological burden. Drug delivery for skin disorders has always been a clinical challenge given that it is essential to achieve maximum possible epidermal penetration and retention with minimum drug absorption into the bloodstream to avoid side effects. Hyper-keratinized skin thickens the cuticle, making it difficult for different active ingredients to circumvent, which directly affects drug delivery and retention. Accordingly, novel drug delivery systems, such as liposomes, niosomes, SLNs, and NLCs hold promise in preclinical and experimental studies, owing to their ability to overcome key formulation challenges.

Many studies have explored this, but further research should be performed. Contemporary research focuses more on polymer and lipid nanoparticles, while metal nanoparticles may attract more interest in future work because of their small size and outstanding performance. Additionally, hybrid nanosystems can combine the advantages of different types of nanoparticles to obtain the most appropriate drug delivery system for patients. This approach has not been thoroughly studied in the local treatment of psoriasis but may greatly improve the management of these psoriatic plaques.

In conclusion, we noted that published data on nano-dermatology appear to be successful at different stages of the healthcare process, offering a personalized approach to immune-mediated inflammatory dermatosis. In the future, high-prognosis psoriasis models (*in vivo* and *in vitro* models) are needed to improve the adoption rates, which may be a key approach in the fight against psoriasis.

## Author contributions

MZ and SH contributed to manuscript writing, editing, and data collection. XS, YZ, YL, LL, JW, CW, and NL contributed to data analysis. XL contributed to conceptualization and supervision. All authors have read and approved the final manuscript.
